# SARS‐CoV‐2 seroprevalence and associated factors among people living with HIV in Sierra Leone

**DOI:** 10.1002/iid3.1338

**Published:** 2024-07-11

**Authors:** Wei Sun, Jinwen Song, Sulaiman Lakoh, Jinquan Chen, Abdulai T. Jalloh, Foday Sahr, Stephen Sevalie, Darlinda F. Jiba, Ibrahim F. Kamara, Yingrong Xin, Zhongyang Ye, Feng Ding, Li‐Zhong Dai, Ligui Wang, Xishui Zheng, Guang Yang

**Affiliations:** ^1^ School of Public Health Southern Medical University Guangzhou China; ^2^ Department of Infectious Disease Control and Prevention People's Liberation Army General Hospital of Southern Theatre Command Guangzhou China; ^3^ Department of Infectious Disease Control and Prevention Chinese PLA Center for Disease Control and Prevention Beijing China; ^4^ Senior Department of Infectious Diseases The Fifth Medical Centre of PLA General Hospital Beijing China; ^5^ Ministry of Health and Sanitation Government of Sierra Leone Freetown Sierra Leone; ^6^ College of Medicine and Allied Health Sciences University of Sierra Leone Freetown Sierra Leone; ^7^ Sustainable Health Systems Sierra Leone Freetown Sierra Leone; ^8^ 34 Military Hospital Republic of Sierra Leone Armed Forces Freetown Sierra Leone; ^9^ World Health Organization Country Office Freetown Sierra Leone; ^10^ Sansure Biotech Inc. National and Local Joint Engineering Research Center for Infectious Diseases and Tumor Gene Diagnosis Technology Changsha China; ^11^ Department of Clinical Laboratory The Fifth Medical Center of PLA General Hospital Beijing China

**Keywords:** HIV infection, immunoglobulin G, SARS‐CoV‐2, seroepidemiologic studies

## Abstract

**Background:**

Human immunodeficiency virus (HIV) infection is an important risk factor for Coronavirus Disease 2019 (COVID‐19), but data on the prevalence of COVID‐19 among people living with HIV (PLWH) is limited in low‐income countries. Our aim was to assess the seroprevalence of the severe acute respiratory syndrome coronavirus 2 (SARS‐CoV‐2) specific antibodies and associated factors among PLWH in Sierra Leone.

**Methods:**

We conducted a cross‐sectional survey of PLWH aged 18 years or older in Sierra Leone between August 2022 and January 2023. Participants were tested for SARS‐CoV‐2 antibodies using a rapid SARS‐CoV‐2 antibody (immunoglobulin M/immunoglobulin G [IgG]) kits. Stepwise logistic regression was used to explore factors associated with SARS‐CoV‐2 antibody seroprevalence with a significance level of *p* < .05.

**Results:**

In our study, 33.4% (1031/3085) participants had received a COVID‐19 vaccine, and 75.7% were SARS‐CoV‐2 IgG positive. Higher IgG seroprevalence was observed in females (77.2% vs. 71.4%, *p* = .001), adults over 60 years (88.2%), those with suppressed HIV RNA (80.7% vs. 51.7%, *p* < .001), antiretroviral therapy (ART)‐experienced individuals (77.9% vs. 44.6%, *p* < .001), and vaccinated participants (80.7% vs. 73.2%, *p* < .001). Patients 60 years or older had the highest odds of IgG seroprevalence (adjusted odds ratio [aOR] = 2.73, 95% CI = 1.68–4.65). Female sex (aOR = 1.28, 95%CI = 1.05–1.56), COVID‐19 vaccination (aOR = 1.54, 95% CI = 1.27–1.86), and ART (aOR = 2.20, 95% CI = 1.56–3.11) increased the odds, whereas HIV RNA ≥ 1000 copies/mL (aOR = 0.32, 95% CI = 0.26–0.40) reduced the odds of IgG seroprevalence.

**Conclusions:**

We observed a high seroprevalence of SARS‐CoV‐2 antibody among PLWH in Sierra Leone. We recommend the introduction of targeted vaccination for PLWH with a high risk of severe COVID‐19, especially those with an unsuppressed HIV viral load.

## 1 INTRODUCTION

In May 2023, the World Health Organization (WHO) declared the end of the Coronavirus disease 2019 (COVID‐19) pandemic, marking a critical step toward ending the COVID‐19 pandemic.^1^ Although COVID‐19 is no longer a global public health emergency, the spread of the severe acute respiratory syndrome coronavirus 2 (SARS‐CoV‐2), the pathogen that causes COVID‐19, is still ongoing.^1^, ^2^ Therefore, in the postpandemic era, countries and regions should establish routine surveillance systems to detect the SARS‐CoV‐2 virus, paying special attention to high‐risk/vulnerable populations, including people living with human immunodeficiency virus (HIV) (PLWH).^2^, ^3^, ^4^ HIV infection is an important risk factor for COVID‐19. Consequently, PLWH experienced adverse outcomes related to COVID‐19 infection, including severe illness and deaths compared to the general population.^5^, ^6^, ^7^

HIV is a public health problem in Africa, with approximately 67% of the global HIV population living in Africa by the end of 2022, with the majority not on treatment.^8^ Sierra Leone, a low‐income country in sub‐Saharan Africa, had an HIV prevalence of 1.7% in 2019.^9^ Despite the low HIV prevalence, the country still faces challenges in the HIV response, such as late‐stage HIV diagnosis and advanced HIV disease.^10^, ^11^ Moreover, due to the challenges in rollout and acceptance, the COVID‐19 vaccine coverage in the general population of Sierra Leone was only 33.5% at the start of the study in August 2022, rising to 52.4% by January 2023.^12^ This rate may be even lower in PLWH. For example, only 17% of PLWH in a tertiary hospital in Sierra Leone received a COVID‐19 vaccine between April and June 2022.^13^

Seroepidemiologic assessment is a powerful method to determine the prevalence of infection, estimate the burden of disease, identify associated factors, and evaluate control and immunization programs. In Sierra Leone, serological data on endemic SARS‐CoV‐2 infection are scarce, and the burden of COVID‐19 remains unknown, especially in PLWH. To our knowledge, only two studies have reported the seroprevalence of COVID‐19 antibodies in Sierra Leone. One national survey in March 2021 reported an overall immunoglobulin G (IgG) antibody seroprevalence of 2.3% and immunoglobulin M (IgM) of 0.6%.^14^ Another study between March and July 2021 reported an IgG/IgM antibodies seroprevalence of 89% among the unvaccinated health facility staff.^15^ As of the time of writing, there are no reports on the seroprevalence of SARS‐CoV‐2 IgG/IgM antibodies in PLWH in Sierra Leone. Understanding the seroprevalence for SARS‐CoV‐2 in PLWH has important policy implications for preventing and mitigating the impact of future public emergencies in this population. Additionally, the findings from this study will add to the global body of evidence on SARS‐CoV‐2 antibodies among PLWH.

Therefore, this study aimed to determine the seroprevalence of the SARS‐CoV‐2 specific antibodies and associated factors among PLWH in Sierra Leone after 2 years of the first confirmed case in the country.

## 2 MATERIALS AND METHODS

### 2.1 Study design and participants

We used a cross‐sectional hospital‐based design in recruiting PLHW aged 18 years or older from high HIV burden health facilities in Sierra Leone. Health facilities with more than 800 PLWH receiving antiretroviral therapy (ART) treatment in Sierra Leone were recruited in the study. However, four health facilities were excluded because of the distance or delay in obtaining consent to conduct the study. Finally, 10 health facilities were included in our study. Participants were recruited using convenience sampling as they visited health facilities from August 2022 to January 2023. Considering the unknown seroprevalence and 33.5% COVID‐19 vaccine coverage rate in the general population of Sierra Leone at the start of the study, we assumed that the overall SARS‐CoV‐2 seroprevalence would be about 40%, with 95% confidence level and 2% absolute error. We then calculated a minimum sample size of 2305 individuals.

### 2.2 Data collection

We consecutively recruited 3127 HIV patients from 10 health facilities in different regions of Sierra Leone. After obtaining written informed consent, demographic, HIV, and vaccine‐related information were collected through a patient interview or a clinical record by trained study staff members using a standardized data collection form to reduce information bias. About 10 mL of venous blood samples were aseptically collected in EDTA vacutainer tubes. We excluded patients with HIV viral load missing and other missing information from the detailed analysis. Therefore, 3085 adult patients were included in the final analysis (Supporting Information: Figure S1).

### 2.3 Laboratory testing

We used a rapid diagnostic test (Innovita) to detect SARS‐CoV‐2 IgM/IgG antibodies by a colloid gold immunochromatography competition assay with a sensitivity of 94.4% and a specificity of 98%, which has been prevalidated and approved to be used in Sierra Leone.

HIV viral load was quantified by real‐time quantitative PCR (Sansure Biotech Inc.) using a Roche lightcycler (Roche Corp.). The detection limit of HIV RNA was 20 IU/mL (2.08 IU/mL = 1 copies/mL).

### 2.4 Statistical analysis

Data analysis was performed using the R software version 4.3.0 (R Core Team, Vienna, Austria). The median (interquartile range [IQR]) and frequency (%) were used to summarize demographic information and HIV and COVID‐19 vaccine details.

We grouped the quantitative variable “age” into categorical variables, labeled as groups 18–30, 30–40, 40–50, 50–60, and 60–100. According to WHO guidelines, HIV viral load was categorized into suppressed (<1000 copies/mL) and unsuppressed (≥1000 copies/mL) groups. We determined the seroprevalence of antibodies as the positivity rate with its 95% confidence interval (95% CI) and Pearson's chi‐square test. Multivariable stepwise logistic regression (backward selection strategy) was used to explore factors associated with SARS‐CoV‐2 antibody seroprevalence and presented as adjusted odds ratios and its 95% CI. The level of significance was defined as *p* < .05.

## 3 RESULTS

### 3.1 Demographic characteristics and HIV details

Demographic characteristics and HIV details are shown in Table 1. Of the 3085 participants, 2299 (74.5%) were female. Median age was 36.0 (IQR 29.0–45.0) years. Up to 2882 (93.4%) were on ART for a median duration of 3.3 years (IQR 1.0–6.6). Among patients receiving ART, 1876 (60.8%) were using the fixed‐dose combination of tenofovir + lamivudine + dolutegravir (TLD). Viral suppression (HIV RNA < 1000 copies/mL) was achieved in 2553 (82.8%) participants.

**Table 1:** Demographic, COVID‐19 vaccine, and HIV details.

Characteristic	*N* = 3085^a^
Age (years)	
18–30	851 (27.6%)
30–40	975 (31.6%)
40–50	702 (22.8%)
50–60	388 (12.6%)
60–100	169 (5.5%)
Median (IQR)	36.0 (29.0, 45.0)
Sex	
Female	2299 (74.5%)
Male	786 (25.5%)
Hospital	
Primary	663 (21.5%)
Secondary	200 (6.5%)
Tertiary	2222 (72.0%)
HIV viral load (copies/mL)	
<1000	2553 (82.8%)
≥1000	532 (17.2%)
ART	
Yes	2882 (93.4%)
No	203 (6.6%)
ART regimen	
TLE	688 (22.3%)
TLD	1876 (60.8%)
Unknown	318 (10.3%)
No ART	203 (6.6%)
ART duration (years)	3.3 (1.0, 6.6)
COVID‐19 vaccination	
Yes	1031 (33.4%)
No	2054 (66.6%)
IgG against COVID‐19	
Neg	750 (24.3%)
Pos	2335 (75.7%)
IgM against COVID‐19	
Neg	2940 (95.3%)
Pos	145 (4.7%)

Abbreviations: ART, antiretroviral therapy; IgG, immunoglobulin G; IQR, interquartile range; TLD, tenofovir + lamivudine + dolutegravir; TLE, tenofovir + lamivudine + efavirenz.

^a^ Median (IQR); *n* (%).

### 3.2 COVID‐19 vaccination

Of the 3085 participants, 1031 (33.4%) received the COVID‐19 vaccine, including Johnson & Johnson (158, 15.3%), Sinopharm (66, 6.4%), AstraZeneca (40, 3.9%), and Pfizer/BioNTech (25, 2.4%) vaccines. The type of COVID‐19 vaccines received by 742 (72.0%) participants were unknown (Supporting Information: Table S1). The median duration of vaccination was 272.0 (IQR 198.5–399.0) days (Supporting Information: Figure S2).

The common reasons for not being vaccinated in the 2054 unvaccinated participants were fear of side effects of the vaccines (1137, 55.4%), the belief that HIV is a contraindication to COVID‐19 vaccination (138, 6.7%), waiting to be scheduled for vaccination (135, 6.6%), no perceived need for COVID‐19 vaccination (64, 3.1%) and worries about the poor efficacy of the vaccines (52, 2.5%). In 391 (19.0%) participants, no reasons were provided (Supporting Information: Table S1).

### 3.3 Seroprevalence

**Figure 1:**
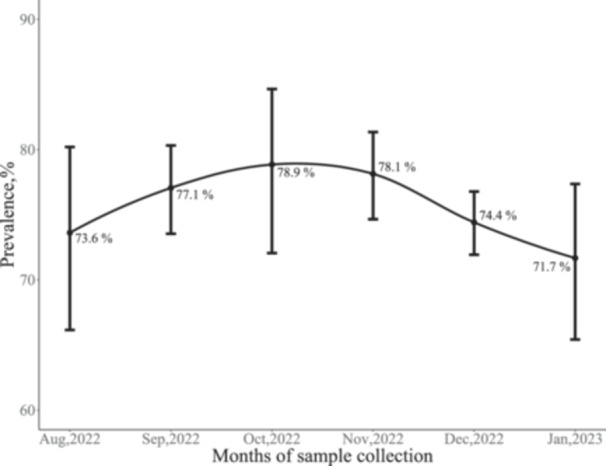
The dynamic seropositivity of IgG antibody against SARS‐CoV‐2 in PLWH of Sierra Leone. IgG, immunoglobulin G; PLWH, people living with human immunodeficiency virus; SARS‐CoV‐2, severe acute respiratory syndrome coronavirus 2.

The overall IgG seroprevalence in PLWH was 75.7% (95% CI, 74.1%–77.2%), whereas few PLWH were positive for SARS‐CoV‐2 IgM (Table 1). The changes in the seroprevalence of SARS‐CoV‐2 during the study period are shown in Figure 1. The IgG seroprevalence was 73.6% in participants enrolled at the start of the study in August 2022. The IgG seroprevalence increased from 77.1% among participants recruited in September 2022 to a peak of 78.9% in October 2022 before declining to 78.1%, 74.4%, and 71.1% for participants enrolled in November 2022, December 2022, and January 2023, respectively.

**Figure 2:**
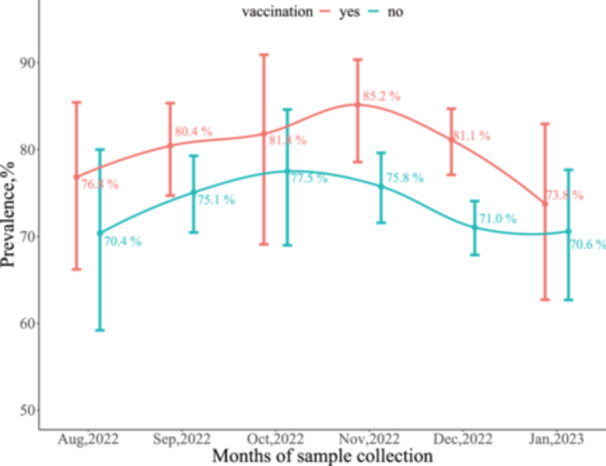
The dynamic seropositivity of IgG antibody against SARS‐CoV‐2 by vaccination in PLWH of Sierra Leone. IgG, immunoglobulin G; PLWH, people living with human immunodeficiency virus; SARS‐CoV‐2, severe acute respiratory syndrome coronavirus 2.

Female participants had a higher IgG seroprevalence than male participants (77.2% vs. 71.4%, *p* = .001). The IgG seroprevalence exponentially increased with age, with rates of 71.8% reported in participants below 30 years, and 75.0%, 77.5%, 77.3%, and 88.2% in those aged 30–39, 40–49, 50–59, and 60 years or older, respectively (Table 2). The seroprevalence was significantly higher among participants vaccinated against COVID‐19 than among unvaccinated participants (80.7% vs. 73.2%, *p* < .001) (Figure 2). Participants with HIV viral load less than 1000 copies/mL had a higher seroprevalence than those with HIV RNA levels ≥1000 copies/mL (80.7% vs. 51.7%, *p* < .001). Similarly, ART‐experienced participants had a higher seroprevalence than ART‐naive participants (77.9% vs. 44.6%, *p* < .001).

**Table 2:** Seroprevalence of IgG antibody against SARS‐CoV‐2 by demographic, COVID‐19 vaccine and HIV status.

Characteristic	Overall (*N*)^a^	Pos, *n* (%)^b^	95% CI^c^	*p* Value^d^
Age				< .001
18–30	851	611 (71.8)	68.6–74.8	
30–40	975	731 (75.0)	72.1–77.6	
40–50	702	544 (77.5)	74.2–80.5	
50–60	388	300 (77.3)	72.8–81.3	
60–100	169	149 (88.2)	82.1–92.4	
Sex				.001
Female	2299	1774 (77.2)	75.4–78.9	
Male	786	561 (71.4)	68.1–74.5	
COVID‐19 vaccination				<.001
Yes	1031	832 (80.7)	78.1–83.0	
No	2054	1503 (73.2)	71.2–75.1	
Hospital				.9
Primary	663	505 (76.2)	72.7–79.3	
Secondary	200	153 (76.5)	69.9–82.1	
Tertiary	2222	1677 (75.5)	73.6–77.2	
HIV viral load (copies/mL)				<.001
<1000	2553	2060 (80.7)	79.1–82.2	
≥1000	532	275 (51.7)	47.4–56.0	
ART				<.001
Yes	2882	2245 (77.9)	76.3–79.4	
No	203	90 (44.3)	37.4–51.5	
Month of sample collection				.2
August 2022	163	120 (73.6)	66.0–80.1	
September 2022	619	477 (77.1)	73.5–80.3	
October 2022	175	138 (78.9)	71.9–84.5	
November 2022	613	479 (78.1)	74.6–81.3	
December 2022	1282	954 (74.4)	71.9–76.8	
January 2023	233	167 (71.7)	65.3–77.3	
Total	3085	2335 (75.7)	74.1–77.2	

Abbreviations: ART, antiretroviral therapy; HIV, human immunodeficiency virus; IgG, immunoglobulin G; SARS‐CoV‐2, severe acute respiratory syndrome coronavirus 2; TLD, tenofovir + lamivudine + dolutegravir; TLE, tenofovir + lamivudine + efavirenz.

^a^ *n*.

^b^ *n* (%).

^c^ 95% CI = 95% confidence interval.

^d^ Pearson's chi‐squared test.

### 3.4 Factors associated with seroprevalence

In multivariable analysis, patients 60 years or older had the highest odds of IgG seropositivity (aOR = 2.73, 95% CI = 1.68–4.65). Female participants (aOR = 1.28, 95% CI = 1.05–1.56), participants vaccinated against COVID‐19 (aOR = 1.54, 95% CI = 1.27–1.86), and those on ART (aOR = 2.20, 95% CI = 1.56–3.11) had increased odds of IgG seropositivity. In contrast, the odds of IgG seropositivity were lower in participants with HIV RNA levels ≥1000 copies/mL (aOR = 0.32, 95% CI = 0.26–0.40) (Table 3).

**Table 3:** Factors associated with a higher seroprevalence of IgG antibody against SARS‐CoV‐2 in PLWH of Sierra Leone.

	Univariate			Multivariate		
	OR	95% CI	*p* Value	aOR^a^	95% CI	*p* Value
Age						
18–30	—	—	—	—	—	—
30–40	1.18	0.96–1.45	.125	1.12	0.90–1.40	.3
40–50	1.35	1.07–1.71	.011	1.20	0.94–1.54	.2
50–60	1.34	1.01–1.78	.041	1.15	0.86–1.55	.4
60–100	2.93	1.83–4.91	<.001	2.73	1.68–4.65	<.001
Sex						
Male	—	—	—	—	—	—
Female	1.36	1.13–1.63	.001	1.28	1.05–1.56	.015
ART						
No	—	—	—	—	—	—
Yes	4.42	3.31–5.93	<.001	2.20	1.56–3.11	<.001
HIV viral load						
<1000	—	—	—	—	—	—
≥1000	0.26	0.21–0.31	<.001	0.32	0.26, 0.40	<.001
COVID‐19 vaccination						
No	—	—	—	—	—	—
Yes	1.53	1.28–1.84	<.001	1.54	1.27–1.86	<.001
Month of sampling collection						
August 2022	—	—	—	—	—	—
September 2022	1.2	0.80–1.78	.358	1.34	0.88, 2.00	.2
October 2022	1.34	0.81–2.22	.258	1.84	1.09, 3.12	.024
November 2022	1.28	0.85–1.9	.222	2.02	1.32, 3.07	.001
December 2022	1.04	0.71–1.5	.827	1.30	0.88, 1.89	.2
January 2023	0.91	0.58–1.42	.670	1.10	0.68, 1.74	.7
Hospital						
Primary	—	—	—	—	—	—
Secondary	1.02	0.71–1.49	.923	NA	NA	NA
Tertiary	0.96	0.78–1.18	.714	NA	NA	NA

Abbreviations: aOR, adjusted odds ratio; ART, antiretroviral therapy; CI, confidence interval; IgG, immunoglobulin G; OR, odds ratio; PLWH, people living with human immunodeficiency virus; SARS‐CoV‐2, severe acute respiratory syndrome coronavirus 2.

^a^ aOR, calculated by multivariable stepwise logistic regression using the backward selection strategy. After variable selection process, age, sex, ART, COVID‐19 vaccination, HIV viral load, and month of sampling collection were finally included in the regression model.

## 4 DISCUSSION

Owing to limited resources, and the lack of scientific research, the true burden of COVID‐19 in Sierra Leone in the postpandemic era is unknown. This study presents data on the seroprevalence of COVID‐19 in PLWH in the postpandemic era.

Overall, we observed that 75.7% of PLWH in Sierra Leone had IgG antibodies against SARS‐CoV‐2. Lower seroprevalence of the SARS‐CoV‐2 IgG antibodies among PLWH was reported in Burkina Faso and South Africa.^16^, ^17^ Although we did not track participants longitudinally, we observed fluctuations in SARS CoV‐2 IgG antibody levels, with a peak level of 78.9% occurring in October 2022 and the lowest level of 71.7% occurring in January 2023. These fluctuations in the SARS‐CoV‐2 IgG antibodies could reflect on the fluctuations in COVID‐19 cases reported in Sierra Leone.^18^

After stratifying the SARS‐CoV‐2 IgG antibodies by age and sex, we observed an increase in the levels of antibodies with age. Young adults below 30 years have lower SARS‐CoV‐2 antibody levels of 71.8% compared to 88.2% in older adults aged 60 years or older. These findings are consistent with the fact that older adults are at a higher risk of SARS‐CoV‐2 infection than young adults.^19^, ^20^, ^21^, ^22^, ^23^ It is widely believed that men are more susceptible to SARS‐CoV‐2 infection and severe COVID‐19 disease than women.^24^ In contrast to this observation, we found that HIV‐infected women in this study were more likely than men to be seropositive for SARS‐CoV‐2 IgG. The reason for this paradoxical finding is unknown but could be investigated in future research.

The IgG seroprevalence among the vaccinated was 80.7% compared to 73.2% among those unvaccinated participants. The high seroprevalence in the unvaccinated participants suggests that the SARS‐CoV‐2 virus may still be circulating in Sierra Leone. This finding should be interpreted with caution, as few PLHW were positive for SARS‐CoV‐2 IgM. On the other hand, the higher prevalence of SARS‐CoV‐2 antibodies in the vaccinated population may be attributed to the hybrid immunity from the natural infection and vaccination. At the end of the study (January 2023), the vaccine coverage (with at least one dose of any vaccine) rate in the general population of Sierra Leone was 52.4%, but the coverage reported in our study is only 33.4% in the same period.^11^ The low number of vaccinated participants who received only one dose of the COVID‐19 vaccine may be due to previously reported vaccine hesitancy among PLWH, resulting in lower vaccination rates.^13^ Given the high seroprevalence of COVID‐19 infection among unvaccinated PLWH and low vaccine coverage, primary and booster vaccinations are still strongly recommended for PLWH in Sierra Leone.

HIV viral load assessment is recommended by WHO and the national HIV program as the preferred method to monitor HIV treatment progress, with a viral threshold of <1000 copies/mL defined as viral suppression.^25^, ^26^ In our study, 82.8% of the participants were virally suppressed, in contrast to a viral suppression rate of 64.6% reported in 2019 and 2020.^27^ It is generally accepted that PLWH receiving effective ART are able to achieve complete or nearly complete suppression of viral replication and maintain immune function comparable to that of the general population.^28^ This statement supports findings from our study, which found that both viral suppression and ART were associated with a higher seroprevalence of SARS‐CoV‐2 IgG antibody after adjusting for demographic factors, vaccine status, and sample collection time.

Our study has strengths. To our knowledge, this is the first national study to determine the seroprevalence of SARS‐CoV‐2 antibody in PLWH in Sierra Leone. The study has a large sample size of 3085 participants included in the final analysis. Furthermore, benefiting from these 6‐month‐long durations of data collection, we were able to identify persistent patterns of fluctuations in the seroprevalence of SARS‐CoV‐2 antibody.

We acknowledge there are limitations of our study. Due to limited laboratory capacity, we only conducted a qualitative analysis of antibodies and were unable to perform a quantitative analysis. We did not establish a general population control group, which prevents us from determining whether the seroprevalence among PLWH is higher. Additionally, our study did not include private hospitals in Sierra Leone, which could be done in the future. Therefore, our results only represent the seroprevalence of SARS‐CoV‐2 antibodies among PLWH in public health facilities.

## 5 CONCLUSIONS

We observed a high seroprevalence of SARS‐CoV‐2 antibodies among PLWH in Sierra Leone between August 2022 and January 2023. We recommend the introduction of targeted vaccination for PLWH with a high risk of severe COVID‐19, especially those with an unsuppressed HIV viral load.

## AUTHOR CONTRIBUTIONS

**Wei Sun**: Data curation; formal analysis; investigation; methodology; writing—original draft; writing—review & editing. **Jinwen Song**: Conceptualization; investigation; writing—review & editing. **Sulaiman Lakoh**: Resources; writing—review & editing. **Jinquan Chen**: Data curation; formal analysis. **Abdulai T. Jalloh**: Writing—review & editing. **Foday Sahr**: Writing—review & editing. **Stephen Sevalie**: Writing—review & editing. **Darlinda F. Jiba**: Investigation. **Ibrahim F. Kamara**: Investigation. **Yingrong Xin**: Validation. **Zhongyang Ye**: Validation. **Feng Ding**: Formal analysis. **Li‐Zhong Dai**: Funding acquisition. **Ligui Wang**: Conceptualization; funding acquisition; writing—original draft. **Xishui Zheng**: Funding acquisition; project administration; writing—original draft. **Guang Yang**: Conceptualization; investigation; writing—review & editing.

## CONFLICT OF INTEREST STATEMENT

The authors declare no conflict of interest.

## ETHICS STATEMENT

Ethics approval was obtained from the Sierra Leone Ethics and Scientific Review Committee (SLESRC) of the Ministry of Health and Sanitation, Government of Sierra Leone in accordance with the relevant guidelines and regulations and declaration of Helsinki. Approval to conduct this study was granted by SLESRC, dated June 21, 2022. Written informed consent had been obtained upon the enrollment of individual participants.

## Supporting information

Supporting information.

Supporting information.

Supporting information.

Supporting information.

## Data Availability

Data are available upon reasonable request.
