# Supplementary material for: SARS‐CoV‐2 seroprevalence and associated factors among people living with HIV in Sierra Leone
Source: Immun Inflamm Dis. 2024 Jul 11;12(7):e1338. doi: 10.1002/iid3.1338 (PMC11238572; doi:10.1002/iid3.1338)
Supplement: Supplementary file 4 — Supporting information. [file IID3-12-e1338-s004.docx]

**Table S1.** Participants vaccination details

|  | **Vaccination, yes**  **N = 1031** | **Vaccination, no**  **N = 2054** |
| --- | --- | --- |
| **Vaccine** |  |  |
| Johnson & Johnson | 158 (15.3%) |  |
| Sinopharm | 66 (6.4%) |  |
| AstraZeneca | 40 (3.9%) |  |
| Pfizer/BioNtech | 25 (2.4%) |  |
| Unknown | 742 (72.0%) |  |
| **Vaccination dose** |  |  |
| One dose | 466 (45.2%) |  |
| Two doses | 406 (39.4%) |  |
| Three doses | 47 (4.6%) |  |
| Four doses | 1 (0.0%) |  |
| Unknown | 111 (10.8%) |  |
| **Reasons for Non-Vaccination** |  |  |
| Afraid of the side effects of vaccination |  | 1137 (55.4%) |
| Worry about the poor efficacy of vaccine |  | 52 (2.5%) |
| Contraindications for the vaccine |  | 138 (6.7%) |
| No perceived need for vaccination |  | 93 (4.5%) |
| Waiting to be scheduled |  | 135 (6.6%) |
| Scheduling conflicts |  | 64 (3.1%) |
| Others |  | 44 (2.1%) |
| Unknown |  | 391 (19.0%) |
